# Developing a *Women's Thought Collective* methodology for health research: The roles and responsibilities of researchers in the reflexive co‐production of knowledge

**DOI:** 10.1111/hex.13804

**Published:** 2023-07-31

**Authors:** Kristen Foley, Belinda Lunnay, Catherine Kevin, Paul R. Ward

**Affiliations:** ^1^ Research Centre for Public Health, Equity and Human Flourishing Torrens University Australia Adelaide Australia; ^2^ College of Humanities and Social Science Flinders University Adelaide Australia

**Keywords:** co‐production, feminism, hermeneutics, knowledge, reflexivity

## Abstract

**Background:**

Co‐produced research holds enormous value within the health sciences. Yet, there can be a heavy focus on what research participants think, do and know; while the researcher's responsibility to explore and re/work their own knowledge or praxis tends to escape from view. This is reflected in the limited use of co‐production to explore broad structural distributions of health and risk(s). We argue this missed opportunity has the potential to unfold as what Berlant calls a ‘cruel optimism’, where something desirable becomes an obstacle to flourishing and/or produces harm. We explore challenges to involving lay populations meaningfully in health research amidst a neoliberal cultural landscape that tends to responsibilise people with problems they cannot solve.

**Methods and Findings:**

Drawing together principles from hermeneutic and feminist philosophy, we develop a novel methodology for co‐producing research about determinants of health and health risk (using a case study of alcohol consumption as an example) that centres on what researchers do, know and think during research: *Women's Thought Collectives*.

**Discussion:**

Keeping the constructed nature of social systems—because they shape ideas of value, expertise and knowledge—in view during co‐produced research illuminates the potential for cruel optimisms within it. Such reflexive awareness carves out starting points for researchers to engage with how social hierarchies might (tacitly) operate during the co‐production of knowledge. Our work has broad utility for diverse population groups and provides important considerations around the roles and responsibilities for reflexive co‐production of knowledge at all levels of health systems.

**Patient or Public Contribution:**

The development of these ideas was sparked by working with lay participants during the Women's Thought Collectives for Kristen Foley's doctoral research 2021–2023, but undertaken without their direct involvement—in accordance with the responsibilities of researchers in the reflexive co‐production of knowledge. Forthcoming publications will address the outcomes and processes of this work.

## 1 INTRODUCTION

It is clear that co‐produced research should happen on topics about health^1^, ^2^, ^3^ and it has become an expected feature of many health‐related research grant applications and funding awards. The benefits of co‐produced research are thought to have value across three axes^4^: (1) it builds relationships with people that live with the issues being researched; (2) develops more robust knowledge to inform interventions that will in turn be more effective or acceptable and (3) upholds an ethical mandate to broaden research participation and knowledge development. Much is written about how variations of co‐production can achieve these values in practice.^2^, ^5^, ^6^ Yet, there remains more focus in this research on what lay populations think and do, rather than consequences for health researchers such as how their values or relationship to the social order must be problematised and changed through these encounters.^7^

Co‐produced research is desirable because it aims to explicate how power and health are unevenly spread across society (according to social hierarchies). Yet, researchers and the systems they work in (including various knowledge ‘economies’) are directly embedded in this unevenness. When the focus of co‐production then is exclusively on what lay populations do (or do not do), it unfolds the potential for cruel optimsm—where something that is desirable because it will promote flourishing, instead, paradoxically prohibits or dissolves it.^8^ When lay populations are only invited to participate in research in ways that fit research/er expectations and environments, it can risk situating them as problematic and dismiss their knowledge (ironically, the very potential for transformation)—while responsibilising them with the burden of solving complex multilevel research issues that require their insight in the first place. Reflexive awareness of the social systems in which health researchers are embedded, including the knowledges which shape perceptions of value,^9^, ^10^ is critical to guard against the unfolding of cruel optimism^8^ during the co‐production of knowledge in health research.

This manuscript discusses a novel methodology that aimed to enhance opportunities for lay women from different walks of life to co‐produce research about health risks, without burdening them with problems they cannot solve: *Women's Thought Collectives*. This methodology was inspired by journalist Ariel Gore's^11^ project to critique happiness through assembling a ‘council of experts’ who informed and reviewed her thinking about western cultural obsessions with happiness. She invited women to think alongside her about how happiness works (or not), when, how, and for whom—akin to Ahmed's^12^ exploration of how happiness gets stuck to different objects and economies to various ends. The council helped collect data and shared their ideas (via keeping a journal), then were invited to contest, extend and corroborate her ideas about happiness over a period of 2 years. This type of co‐production methodology could expand investigator knowledge over time while also giving participants space and time to adjust and inflect interpretations. Her book included limited methodological details, so rather than following her approach, we attempted to develop our own, although loosely based on her council of experts.

The flipped positioning of this approach places the researcher's learning, thinking and reflections in view and open to exploration by research participants. We explored how these ideas sat with key principles from hermeneutic and feminist philosophy, then developed a novel methodology for Kristen's doctoral research, which focused on illuminating the commercial determinants of alcohol consumption for Australian women in midlife. The purpose of our paper is fourfold.

1. Reflect on the flipped positioning of a council of experts methodology, which brings into view what researchers think, do and know (reflexivity) when co‐producing research in health settings
2. Explore how contemporary feminist and hermeneutic philosophical thought might add to, or refine, methodologies for co‐producing health research
3. Outline the *Women's Thought Collective* methodology which will be of use and interest to researchers in diverse health settings
4. Discuss how to pursue co‐production research that *does* elevate the role of lay populations in research but *does not* reconstitute a cruel optimism by eliding roles and responsibilities for researchers during the co‐production of knowledge.

We, therefore, distill key ideas from hermeneutic and feminist philosophy, then reflect on critical ethical considerations for health researchers to keep in mind when co‐producing research in neoliberal settings—a cultural landscape that tends to reconstitute the problem and responsibility for solution^8^ within groups and individuals already experiencing oppression. We hope that this structure will showcase the flow of ideas from the macro context of philosophy (presented in the methods) into the specific methodology developed (outlined in the findings) and residual questions (raised in the discussion) for co‐produced research about upstream distributions of health resources and health risks.

We weigh co‐produced research against its potential to enable different groups to exert control over knowledge as a resource and determinant of their lives and living conditions.^13^ This focus on power and social structure illustrates the democratic and politically‐motivated nature of co‐produced research and demarcates it from ‘cobiquity’ where broad and diffuse research partnerships might constitute co‐production.^14^ Aligned with the ‘values and principles’ typology of co‐produced research, differences between researchers and lay experts are democratised, and the co‐production process is treated as evolving rather than a rigid or fixed concept.^2^ We recognise there are diverse histories and disciplines which inform the words used to describe involved participants in co‐produced research, and that rather than these terms (i.e. lay, expert, research participant) having objective meanings we argue they are made meaningful through their contextual relationship to the researcher/s and research process/es. In the co‐production sphere, a key feature of this contextuality can include whether participants are paid and how (much), as well as what is possible in the cramped spaces of academia.^15^

While these terms matter, we use several within this manuscript but do not see this to contradict our central argument: that keeping the contextuality in view enables exploration of how health researchers, lay knowledges and co‐production initiatives can be situated around socio‐political structures including historical trajectories and the distribution of power and resources^16^—the key undercurrent of co‐production research. Our research context is a useful area for this, in exploring *alcohol* consumption (a health risk in a healthiest society that carries sociomoral dangers^17^) by *women* (who have historically been scrutinised, surveilled and muted) during the multilayered transitions of *midlife* (culturally constructed in the west around erasure and invisibility^18^). In Australia, alcohol is complexly interwoven with social life and empirical evidence shows it has significant use‐values for women in midlife; including managing uncertainty,^19^ happiness,^20^, ^21^ and wellness^22^ as well as smoothing difficulties at home.^23^

### 1.1 Co‐production research in health: All about what participants do?

Early arguments for involving lay populations in health research responded to the recognition that they hold expertise that will enhance understanding of health and its social genesis.^1^, ^24^ The distinct, plural and free‐form ideas from lived experience are now seen as critical to realising epistemic justice within democratic methods,^25^ yet this sits in tension with ingrained logics of natural science which pursues the exclusion of participants and their subjectivities.^26^ It may also irritate tightly wound socialisation(s) around the authority and expertise of health professionals and systems, to the extent that both lay experts and health researchers tend to misinterpret and downplay what and how to lay populations can contribute to health research.^9^ These knowledge contests are scaled up during power‐focused co‐production methods,^5^ because they explicitly seek to elevate the power participants have in research processes and even help to reimagine^27^ and reorganise^28^ power dynamics. This radical politic likely underpins why turbulence will unfold if co‐production is done ‘properly’.^29^

Researchers are directly embedded in these dynamics, which is why reflexive practices are so central to^30^, ^31^ and entangled within knowledge systems, prompting our focus on epistemic^9^ and ontoepistemological reflexivity.^32^ The perceived value of knowledge moulds subjectivities,^10^ and then influences what research participants think to disclose and what health researchers think to ask during research encounters.^9^ Unasked questions and undisclosed knowledge are key examples of the need for reflexivity and showcase its relationality with epistemic privilege.^33^ This is crucial within co‐produced health research, where despite its political ambitions, lay participants are less commonly involved in healthcare planning, design, delivery and improvement^34^ or the review of systems innovation.^35^ Instead, they are more typically represented in technical aspects^36^ of research more acceptable in neoliberal settings but still branded as co‐production, such as activities like usability testing that can be more passive and defined because they have a preset agenda.^37^ Concomitantly, theoretical guidance about how to involve lay populations in co‐analysis of data has been obscure^38^ with some suggestions that these processes need ‘downgrading’^39^ or risk being ‘dirtied’ and ‘stretched’ during co‐production.^6^ Lurking doubts about the capabilities of citizens to engage in co‐produced research^40^ alludes to lingering conflicts about whose knowledge is legitimate and in what settings. How knowledge is valued further influences what is viewed as a valuable output from analytical or interpretive processes—further showcasing why the histories that give rise to contemporary configurations of knowledge are manifestations of power that enfold co‐produced research.^16^

Broadly, the potential role—and value—of involving lay populations at macrostructural levels of in/equality remains undertheorised and underachieved.^16^ We suggest that there are two layers to this problem. First, the value of lay expertise might be misunderstood (*a reasoning error*) and therefore efforts are not invested in developing researcher critical consciousness towards epistemic and political contests. The fact that much of what citizen scientists produce is ignored by scientists^41^ is likely symptomatic of this, in addition to unwritten rules about whose expertise matters and whose does not.^42^ Second, the short‐term and scarce nature of health‐related funding and implementation cycles contracts the scope for such work (*an execution error*) because it does not afford the time and care needed for collaboration^43^ that is sustained, open‐ended, and able to transform civic arenas.^44^ Both of these co‐production ‘errors’ engender layers of cruel optimism.

The *reasoning error* continues to situate lay participants as the *problem*, in a position of deficiency where they *lack knowledge* (per Foucault,^45^ docile bodies that are positioned as vacuous vessels that need filling with new information)—rather than the knowledge they bring being examined by researchers to break cycles of ‘circulating reference’.^46^ It then follows that they cannot bring air into closed political systems^15^ nor disrupt the quest for monophonic patient voice in health settings^47^ because the ways in which the knowledge they bring can be valued is already prescribed by dominant cultural systems that privileges some knowledge above others.^10^ The *execution error* occurs when the value of lay participants is understood (at least to some level) but the expectations, needs and practices of co‐production research spill over what health researchers can fulfil—resulting in disengagement (and/or moral injury on behalf of the researcher and participants)—as well as cramping of what can be achieved during the process,^15^ which turns the gaze again to what lay participants do, think and know that can be made available for critique in a short‐term window.

Considering that epistemic precarity frequently coincides with material precarity,^48^ the cruel optimism of co‐production can be twofold—rhetorically and materially engaging with lay populations who experience disadvantage *because* of this perceived vulnerability while dissolving potential benefit from their involvement, because their insight is not viewed as ‘knowledge’ nor enabled to challenge the ‘status quo’ of knowledge production systems.

## 2 METHODS

### 2.1 Research context and prelude to women's thought collectives

In thinking about how to transition the snippets of Ariel Gore's council of experts method detailed in her book (as we were unable to connect with her in‐person), she talks about travelling to meet different women she knew or contacted because they had expertise in exploring the question of happiness, drawn either from lay or professional experience. We liken her approach to purposive sampling in an emergent research design,^49^, ^50^ where she included people at unstructured points throughout her project in response to what she learned. Gore^11^,^p.9^ states wanting to ‘ask women and femmes of all genders – groups who'd been extensively studied when it came to depression and completely ignored when it came to positive psychology – what made them happy’. After interviewing hundreds of women (p. 22), she then ‘convened a council of experts – artists, mothers, service workers, scholars, psychologists, and women's health‐care providers… to keep their own journals for several weeks and then join me for a liberation psychology forum’ (p. 22, number of women within the council unstated).

The value of this open‐ended approach was identified as an innovative way of centring the researchers' growth and reflection (in contrast to lay participants) during Kristen Foley's doctoral research exploring the commercial determinants of alcohol consumption in the context of rising consumption levels for Australian women aged 45–64 and associated increases to population breast cancer risk.^51^ A linked study was already running within the public health department in which Kristen's PhD would be located, to explore how social class shapes alcohol consumption for women in midlife using interview methods^52^. To complement these insights, she designed and received funding for a companion study to explore the social context of alcohol consumption and experiences of marketing to women to increase profit margins and market share. Given that so much of targeted marketing is specifically tailored and delivered to particular groups and individuals to increase purchasing and consumption, involving the lay population seemed unavoidable and completely necessary.

The timely and evocative reading of Gore's^11^ book, however, sparked creativity about how research participants could be involved in co‐analysing and co‐interpreting research findings about them—co‐producing knowledge about issues that concern them while also placing them in a position to refine the evolving meanings and interpretations made from the research. There were few details, however, about the liberation psychology forum she held and it was unclear how her ideas were refined by the presence of her council of experts (hence our reason for contacting her). This move from what was said to her, her reflecting over what it meant and then pulling into a narrative—makes stark what is done to knowledge between its ‘provision’ by research participants and subsequent ‘development’ by researcher(s) during analysis and interpretation, typically without recourse to participants (member‐checking being an exception, although still subject to the researcher terms of conduct). Something Gore captures well is her wrestling with these ideas, both in granular detail and how it changes her ideas about what happiness is. She journals about trying to live differently according to the knowledge she adopts. In using her work to spark intellectual creativity,^53^ we set out to explore how a council of experts might resonate with ideas in the social science literature about co‐producing research regarding structural distributions of consumption, health, risk and resources.

### 2.2 Developing insights from contemporary feminist and hermeneutic philosophy

We summarise here the learning from (1) hermeneutic and (2) feminist philosophy, which were read in response to Gore's^11^ method to ground it in the contemporary thinking about knowledge, truth and method—and infused a co‐production methodology to create space for researcher reflexivity under the guidance of co‐analysis and co‐interpretation of lay participants.

### 2.3 Hermeneutic philosophy

Philosophical hermeneutics explores in‐depth what it means to ‘know’, ‘understand’ and ‘interpret’.^54^ Centrally, hermeneutic thinkers are convinced that our being and essence as humans is to *interpret* (following Heidegger^55^) and that we *are* what we know, which comes from understanding^56^ and feeds back into new knowledge.^57^ In this conceptualisation, knowledge is not something special cordoned off in the realms of science or literature. Rather, we live it in everyday life through what we do,^54^ whether this be conscious or not and this is how humans transmit the effects of history.^58^ Given how knowledge is shaped by early learning, there can be no ‘absolute zero’ to knowledge^59^; rather through our lives, we embody different facts which come from both private and professional spheres.^60^ Because some of this happens unconsciously, hermeneutic thinkers argue that we can never ‘bracket out’ bias from the research process (as assumed in positivism) because we do not know what effect it has.^54^ Instead, hermeneutic methods aim to build a positive relationship with prejudice^54^—by turning inwards, to the self, uncovering how what we think and do is driven by particular ideas and beliefs, and exploring what angles are opened up through this as a field of learning.^61^ This is akin to the practice of reflexivity, although it takes a particular focus on the ontological (nature of being) and epistemological (nature of knowledge) level.^32^

The researcher and their reflexive posturing is therefore as much the object of study in hermeneutics as is the research topic or population of interest. This focus is brought to life by the use of a knowledge horizon as imagery to picture hermeneutic methods. When looking at a horizon, the objects in the foreground shape what is seen on the horizon. As the position of the researcher shifts—they get a different view of the foreground objects and also the horizon^61^—this metaphor emphasises that where the researcher ‘stands’ influences what they ‘see’. Recognising the positioning of the self in generating knowledge is therefore quintessential to working with hermeneutics^32^, ^62^, ^63^, ^64^ and a hermeneutic situation can only be said to have been achieved if there has been a change in the researcher's way of understanding the world.^65^ In terms of thinking about how hermeneutic ideas might be useful in co‐production research, because there is no possibility of complete knowledge^66^ the research project is attuned to the context(s) in which these views arise. Methods would be thought less about as processes for data gathering and more about ways of becoming entangled with how things work (or not) for different people in the world.^67^

### 2.4 Feminist philosophy

Feminist philosophy is centrally concerned with exploring how gendered power moves in and through everyday life. The concept of gender was constructed to enable women to be considered outside of the relationship to their reproductive body alone,^68^ and contemporary feminist methods seek to understand how aspects of gender intersect complexly^69^ with other identities/experiences of exclusion or subordination.^70^ Feminist historians in Australia have traced how exclusion from commanding economies has been linked with madness,^71^ the ways in which social demography furthered control of class and family politics in Australian colonies,^72^ and also the invisible power and privilege of whiteness within Australian feminism.^73^ Feminist philosophy is strongly linked with activism, where a diverse range of theoretical positions^74^ explores whose knowledge has counted^75^ through diverse methods that loosely cohere around centring lived experiences^76^—particularly those excluded from dominant societal narratives.^12^, ^33^ Feminist theory and research can therefore be thought of as a disruptive knowledge practice^77^ that aims to unsettle traditional power differentials around knowledge production, in which researcher reflexivity^78^ and positionality are key.^79^

Health systems and sciences have often used an extractive or possessive logic for knowledge acquisition,^80^ which tends to erase the sociopolitical context from which knowledge is developed^81^ while shedding the relationships which gave rise to it.^80^ By contrast, feminist methodologies aim to centre relational methods of knowledge production and theorise the social world (and degrees of epistemic privilege) in relational ways.^33^ Considering the historical positioning of the female body as deficient in much of medical and health discourse/practice,^82^ the health system is an important landscape in which giving voice to women's experiences and how women understand themselves^83^ takes on political and ethical significance.

## 3 FINDINGS: *WOMEN'S THOUGHT COLLECTIVES* AS METHODOLOGY

The methodology involved two different groups of women being recruited to oversee Kristen's doctoral research over a 2‐year period, with the invitation to co‐collect research data and contribute to thinking through research data and findings with the researcher. The term ‘oversee’ was used specifically to signpost the elevated role of women within the research methodology, beyond what is typically conjured when thinking about a ‘research participant’ (contributing data only) – as well as illuminate the gaze on the researcher. The key principles of the methodology were:

1. Elevating the role of lay researchers in developing knowledge that concerns them, without responsibilising them with problems they cannot solve.
2. Opening as many opportunities for researcher reflexivity and accountability through data co‐collection, co‐analysis and co‐interpretation—and recognising this is where the legwork and insight should emerge.
3. Being explicit with participants about research is one way of knowing amongst many, including that gained from everyday (and a lifetime of) living, thinking and doing.
4. Adopting a flexible research approach that enables change to methods based on input from involved participants.

**Figure 1:**
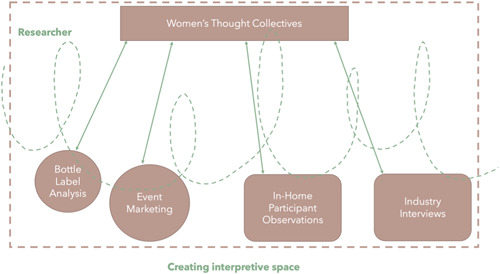
Women's thought collective methodology.

These principles are represented in Figure 1, where the spiral line shows how the Women's Thought Collective could inform, influence and reflect on the studies designed to comprise the doctoral research before recruiting them as a Thought Collective (due to funding cycles running so far ahead of the work starting). The novelty of the methodology is that the Collective was engaged throughout the research process to inform decision‐making and reflection, but the gaze is focused on what the researcher is doing and how their knowledge (and research progress) shifted in relation to reflections contributed by the Collective. We decided to use the term Women in the Thought Collective title (as an attempt to deter women from participating in the case it was named ‘expert’, etc.).

### 3.1 Methods

Two Collectives were assembled; one with midlife women from South Australia and another with midlife women nationally. The state‐based Collective was where the researcher was located and also where the parallel study had run about the reasons as to why women in midlife drink^20^, ^52^, ^84^ which could be explored in relation to broader structures, the focus of this study. The national Collective was assembled to help elucidate differences between states and create a further reflection on the experiences and perspectives of women living in different locations. Group meetings and individual conversations were combined in a flexible, semistructured process, on the logic that both contributed to creating entry points for reflexivity for the researcher. Three group meetings were held with each group (*n* = 6) at the beginning of the doctoral work to discuss ideas for exploration which had emerged from gaps in (1) the parallel sister study; (2) the multidisciplinary literature review regarding commercial determinants of alcohol consumption and (3) the extent to which the proposed design would achieve an understanding of these gaps. Research participants were provided with journals and asked to record any instances of alcohol advertising or commercial activity they saw, thoughts about how expectations or practices around alcohol had changed across their lives, views on alcohol companies, and reflections on work, play and rest generally as they had grown and transitioned through the life course. They were encouraged to email any photos or reflections in real time to the researcher. The researcher, in turn, also kept a regular reflexive research journal that documented reflections about input from collective members, the research process and emerging thoughts drawn from engagement with academic literature.

### 3.2 Recruiting (and describing the research)

Significant thought was put into how to convey the value of lay expertise within the research to participants, with recruitment material outlining what participation would involve:

1. Recording in a journal observations and reflections about alcohol consumption and relevant advertising/marketing in your own life.
2. Attending 4–5 meetings with ∼15 women throughout 2021–2022 (scheduled on demand in relation to what is coming out of the research) to discuss your reflections as well as data analysis and interpretation in the wider study, which will be audio‐recorded (for purposes of note‐taking and background analysis only).
3. Providing input and guidance to Kristen during the development and progression of ideas surrounding alcohol consumption for Australian women in midlife.

Interested women were encouraged to contact the researcher. The following script was used to convey to them why participating for such a long period of time was useful, per Box 1:

Box 1 The idea for holding a Thought Collective came about because while many people participate in research, they do not have a role in influencing the analysis, outcomes or interpretation of that research. We hope the Thought Collective will engage women directly in developing knowledge that concerns them.

We distributed this material around university networks (i.e., flyers) and relevant community groups (i.e., notice boards and by contacting specific groups). Snowball sampling was also used, where motivated participants contacted others to suggest participation. Those who participated were provided reimbursement for their time and participation. We aimed for as much heterogeneity in the sample as possible, including in living circumstances (i.e., single/married/divorced, having had children or not, having elderly parents, living alone or with others, in metropolitan or regional areas) and social class (a mixture of economic, social and cultural capitals as measured by a validated tool^85^; drinking level (light, medium or heavy as measured by the AUDIT‐C tool) and age range between 45 and 64.

We did not target participation from any Aboriginal or Torres Strait Islander communities, recognising that Australia's colonial legacy shapes alcohol practices in ways that differ for Caucasian women in midlife.^86^ No women communicated identifying as Aboriginal or Torres Strait Islander. We recognise the paradoxical nature of arguing that disadvantage accumulates at layers of intersections and the knowledge they bring is critical to unsettle entrenched hierarchies and power dynamics^27^ but not specifically targeting groups likely to have diverse ontoepistemological positionings.^33^ Following our logic of widening participation in knowledge development, draft outputs will be circulated to a wide range of audiences, that accounts for groups (i.e., organisations or champions) that may not have been represented in the Collective and could be used to develop future partnerships if desired by these groups. This will create further entry points to co‐producing (or contesting) knowledge, and aligns with Erikainen et al.^79^ support for widening the way in which ‘research engagements’ are conceptualised and the structural conditions under which they occur.

### 3.3 Undertaking the research

**Figure 2:**
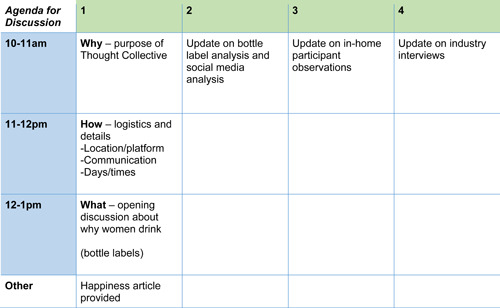
Agendas for thought collective methodology.

The Collectives were shown Figure 2 at the first meeting to try and offset any feelings their knowledge was not valuable and might not influence the trajectory of the project—by way of blank spaces being included in the plan, contingent on forthcoming discussions. We also asserted that the purpose of the Collective was not to come to a consensus about an issue, because such a task was unlikely and intractable—rather, to unearth a range of different perspectives and how these all might help to understand the commercial determinants of alcohol consumption for Australian women in midlife. This openness to dissent and difference is important for co‐produced research so that perspectives are not marginalised nor recast through hegemonic values needed to present uniformity or ‘one voice’,^87^ ideas contested by democratic methods which highlight the problematic nature of conflict‐free consensus such as agonistic pluralism.^88^ We provided examples of the breadth and depth of information that might have been relevant—a bottle label, in‐store motivators for purchase, etc.—to make what we were hoping for seem approachable and clearly tied to their expertise of what happens to them in everyday life.

The vision of the methodology was designed to prioritise lay participant input for analysing and interpreting research findings and outcomes, while the legwork for doing so forces the researcher's decision‐making to remain in view throughout the study. Accordingly, 1–2 page summaries were developed for each research activity undertaken and sent to members of the Thought Collective to act as feedback windows into the development and circulation of ideas throughout the project. This also kept them engaged with the research and maintained a relational or reciprocal element. The researcher maintained a progress sheet of contacts to ensure that each participant member was checked in with at regular intervals, and sent a snippet of something that might spark conversation or for them to see something in their lives differently. Regular and ad hoc communication was typically by email, with frontloading that a nonresponse would be interpreted as them being too busy at the time, and would be re‐contacted again in a few weeks—but that they could cease their participation if they asked via return email. Participant preferences were followed regarding communication, and recognising that some preferred phone contact, emergent thoughts and ideas were discussed with participants via phone rather than in addition to email.

Field notes were taken throughout the project. This resulted in reflections about different ontologies and epistemologies held by the participants in the Thought Collective, which helped to understand the research topic (commercial determinants of alcohol consumption), spark reflection for the researcher about their own ontoepistemological positionings, and create space for problem‐solving any ideas raised by participants. An example is included below in Box 2:

Box 2 One of the Collective members suggested we explore with police what data they have available about women in midlife being fined for drink‐driving because she had a few friends who drove only because their partner was too drunk to drive (male in her examples). I did this twice in 2021 via the general inquiry line through police, but received no input. I've been thinking about it again, as to whether I should pursue it, but was just thinking today how I don't think I viewed it as important as other lines of exploration because it's not as clearly linked to commercial determinants of health—although of course, lay participants do not see their lives in such terms, and lines of gendered power are a key way in which structures (which in our modern life are significantly influenced by actions within the commercial sector) are lived by people. So—it is highly relevant, and I'm embarrassed I didn't follow it up more aggressively earlier on. I'm going to try and pursue a different line of inquiry, to get in touch with an academic that has published a few pieces in partnership with the police, to see if they can help with pursuing this line of inquiry further, even though I'm not sure where it will go.

In this excerpt, it is the researcher who changes over time evident in the reflexivity here about ontologies (as influenced by consumption and commercial activity) and epistemologies (where ways of knowing are highly specialised for researchers and can variously inform the question of relevance, which are linked differently for lay participants). The change came about in response to the ideas of a Collective member and the consideration of philosophical concepts. Another reflection in Box 3 surrounds the tendency towards missing bodies in descriptions and discussions about alcohol:

Box 3 It is curious to me that often I can have a whole conversation with someone about alcohol (work‐related or not), or read a whole article about alcohol, and what the body does can be absent from them… it makes me think about Elizabeth Grosz's ‘volatile bodies’ argument, where bodies were overlooked as orienting or disrupting forces in the philosophical ideas prominently circulated in the west… and bridges to some of the reflections from Collective members that talked about midlife as being a time where they had to (or were able to) pay attention to their bodies in ways they hadn't earlier in their lives or for some period of time. I wonder if alcohol has a role in facilitating this awareness?

Time passing was important to being able to have these discussions with participants and a key source of benefit to the novel insights which can emerge when what the researcher does, thinks and knows is brought into view during co‐produced research. The attention to this space for reflexivity also means that questions of method are clearly in view and considered for discussion throughout the research, and has led to the researcher reading in ways that support ongoing ontoepistemological reflexivity (i.e., privileging reading texts from authors with life experiences and worldviews different to the researcher).

## 4 CRITICAL CONSIDERATIONS FOR HEALTH RESEARCHERS DURING THE CO‐PRODUCTION OF RESEARCH IN NEOLIBERAL SETTINGS

Clear synergies exist between hermeneutic and feminist praxis: in both, the researcher is embedded and entangled in knowledge production, to the extent that they cannot be separated from it.^33^, ^78^ Health researchers become subjects of inquiry, rather than objects/objective parties in inquiry, messying the power dynamics of traditional research^89^ because it becomes the researcher's reflexive ability to recognise, make available for comment—to lay participants and research audiences—how they will consider the knowledge that is objective, subjective or emergent in local worlds.^5^ This flipped expectation is evident in our *Women's Thought Collective* methodology, where the researcher must reflexively work between their own values and the knowledge or value systems these are fostered by^10^ to co‐produce knowledge about health. The view that knowledge is a social commodity developed out of science, health *and* lived experience must become part of expected literacy and discussion for health researchers working in co‐production. It could be embedded into training and education with health professionals but also publishing expectations; perhaps the mandated inclusion of a section within manuscripts where researchers reflect on what they will do, think or know differently as a result of research that involved lay participation (broadly defined). Ensuring that researchers are held responsible for attention to scientific quality^90^—where this involves a working knowledge of the philosophy of co‐production methodologies as well as the research methods they might use, will be critical during editorial and peer‐review processes.

We agree that co‐production requires agreement on principles and values rather than definitions,^2^ and suggest that when principles that illuminate the democratic and political undercurrents necessary for productive co‐production research are outlined,^14^ reasoning and execution errors may be lessened. The value of genuinely involving lay participants in research must be coupled with a wariness of co‐production as a new buzzword of neoliberalism^34^, ^91^ that can engender complicity in structural inequalities^92^ during co‐produced research that apparates the researcher and the knowledge systems in which they work within out of the picture. Centring the difficulty of this work may help to further guard against these errors in co‐produced research and glossing over the dislocation and disorientation during forms of public deliberation.^93^ Our work adds to crucial reflection about co‐produced research which can scapegoat lay participants during ‘inefficient’ co‐production research^89^ and risk a twofold cruel optimism, which not only impedes flourishing but also diminishes it.^8^ By contrast, we present a methodology that situates the researcher as responsible for cohering co‐production methods that take form around socio‐political structures and the distribution of power and resources,^16^ by attending to the contested value/s of knowledges. The philosophical view of entangled knowledge production—drawn from feminist and hermeneutic domains, and enacted through reflexivity—illuminates the potential for cruel optimisms within co‐produced research while also outlining pathways to guard against them. Attending and responding to these entanglements are critical roles and responsibilities of health researchers during the reflexive co‐production of knowledge because they stem from the epistemic origins of entrenched inequalities in health research that necessitates the participation of lay populations in the first place.

## AUTHOR CONTRIBUTIONS

Kristen Foley designed and undertook the Thought Collectives, including securing grant funding, organising ethics approvals and recruiting participants. She conceived and wrote the manuscript. Belinda Lunnay substantially contributed to the design and facilitation of the Thought Collectives as well as adjusting the design, ethics requirements and possibilities for interpretation in live circumstances. She critically reviewed the manuscript for important intellectual content. Catherine Kevin substantially contributed to the design of the Thought Collectives as well as an approach to data collection, analysis and interpretation. She critically reviewed the manuscript for important intellectual content. Paul R. Ward substantially contributed to the design and facilitation of the Thought Collectives, including support of grant and ethics applications. He reviewed the manuscript iteratively for important intellectual content.

## CONFLICT OF INTEREST STATEMENT

The authors declare no conflict of interest.

## ETHICS STATEMENT

Data cited in this paper are drawn from field notes from Kristen Foley's doctoral research, which has ethical approval from Torrens University Australia.

## Data Availability

Data involved in developing this manuscript can be made available from the authors upon reasonable request.
